# Pregnant women’s health beliefs about pre-eclampsia in a resource-limited setting: A qualitative study

**DOI:** 10.1371/journal.pone.0361626

**Published:** 2026-10-30

**Authors:** Isabella Garti, Awube Menlah, Emuejevoke Fuafugo, Evans Osei Appiah, Nadyen Jordan Shikpup, Ophelia Serwaa Tonto, Betty Osei Ntiamoah

**Affiliations:** 1 Molly Wardaguga Research Institute, Charles Darwin University, Darwin, Northern Territory, Australia; 2 School of Nursing and Midwifery, Valley View University, Accra, Greater Accra Region, Ghana; 3 National Orthopedic Hospital, Lagos, Nigeria; 4 School of Nursing, Purdue University, West Lafayette, Indiana, United States of America; 5 School of Nursing, University of Jos, Jos, Plateau, Nigeria; 6 Zenu Polyclinic, Ghana Health Service, Kpone Katamanso, Ghana; 7 Maternity Unit, Ashanti Regional Hospital, Kumasi, Ashanti Region, Ghana; University of Gothenburg: Goteborgs Universitet, SWEDEN

## Abstract

**Introduction:**

Pre-eclampsia, a high-risk pregnancy complication, poses significant threats to maternal and fetal health. Globally, little is known about how women navigate a pre-eclampsia diagnosis or their perceptions of the pathology, unpredictable course, and treatment. Guided by the Health Belief model, this study explores pregnant women’s beliefs about pre-eclampsia in Ghana’s most populous region.

**Materials and methods:**

A qualitative descriptive study was conducted at a district hospital in the Ashanti Region of Ghana to explore pregnant women’s narratives of their health beliefs and perceptions regarding pre-eclampsia. Sixteen participants were purposively selected, and one-on-one semi-structured interviews were conducted in October 2021. Data were analyzed using qualitative content analysis. Categories were developed inductively from participants’ accounts and subsequently interpreted in relation to the constructs of the Health Belief Model.

**Results:**

Pregnant women’s risk perceptions of pre-eclampsia varied, with some not perceiving personal risk, while a few felt vulnerable based on community experiences and observations of other women. Pre-eclampsia was widely regarded as a serious condition, with many expressing fears of adverse maternal and fetal outcomes. Participants recognized the benefits of antenatal care and lifestyle modifications in reducing risk and empowering care-seeking, while previous experiences with pre-eclampsia and guidance from health providers served as key cues to action. However, several barriers to care were identified, including financial constraints, mistrust of the health system, and inadequate communication from health providers.

**Conclusion:**

This study highlights the need for context-specific approaches to address health beliefs related to pre-eclampsia. Strengthening maternal health literacy, effective risk communication, and respectful care can improve early recognition, care-seeking, and outcomes for pre-eclampsia.

## Introduction

Pre-eclampsia, a high-risk pregnancy complication, poses a substantial risk to maternal and fetal well-being [1,2]. The complication carries a 20-fold risk of maternal mortality and subsequent lifelong effects [3]. In developing countries with limited maternal healthcare, pre-eclampsia is a leading cause of maternal deaths [3,4]. The multifactorial nature of pre-eclampsia pathogenesis, along with its unpredictable progression and risk for deterioration, poses a challenge for management [4,5]. Hence, early detection and targeted life-saving interventions, such as referrals and adequate treatment, play a major role in improving outcomes for the mother and fetus, especially in low-income settings [5,6].

Risk perceptions refer to an individual’s belief about how likely they are to experience a health threat [7]. Risk perceptions are multidimensionally embedded in social value systems, physical circumstances, and organizational factors [8–10]. The risks perceived in maternity care, especially during a high-risk pregnancy, are also multifaceted and influenced by factors such as cultural background, past experiences, and inadequate knowledge [8,11–13]. Pregnant women with social disparities who develop high-risk conditions are also more vulnerable and may have a poor risk perception of their situation [14]. Previously, it has also been documented that these varied determinants influence women’s perceptions of risk, making them skeptical and non-compliant with health information and suggested behaviour modification [10].

In the context of clinical care, health professionals and pregnant women often have contrasting views and perceive risks about maternal high-risk conditions differently, leading to tensions that have implications for care and pregnancy outcomes [13,15,16]. These differing risk perceptions can directly shape care-seeking, communication, and decision-making during pregnancy. When women underestimate risk, they may delay or avoid care. Conversely, when health professionals emphasize biomedical risk without addressing women’s lived experiences and concerns, it can lead to mistrust, poor communication, and disengagement [17]. Misunderstandings regarding pre-eclampsia and eclampsia are documented [18–20]. In one study, women’s perceived susceptibility and threat of pre-eclampsia and eclampsia influenced their care-seeking actions [12]. Therefore, adequate knowledge and heightened risk perception on the part of both the clinician and the recipient of care are necessary to optimize outcomes in pre-eclampsia management.

Ghanaian pre-eclampsia research leans heavily on the biomedical aspects, disease trends and adverse outcomes [21] management strategies and care practices [22,23], whilst the woman component has been left out of the loop. Only a few published studies have examined levels of knowledge among women, and even fewer have explored women’s perceptions, leaving a significant gap in the literature [18,24]. To bridge this gap, this study aimed to explore and describe pregnant women’s health beliefs and perceptions of pre-eclampsia in Ghana, as this understanding is essential for improving the quality of care for women who develop pre-eclampsia.

## Materials and methods

### Study design

A qualitative descriptive approach [25] was used to explore women’s perceptions within their real-life context without requiring high levels of abstraction. Data were examined through qualitative content analysis using a hybrid inductive–deductive approach [26,27]. The analysis began inductively, allowing subcategories and categories to develop from participants’ accounts [26]. The Health Belief Model (HBM) [28] was then used as a deductive lens to interpret how those categories related to its constructs. This sequence allowed participants’ experiences to shape the findings before they were considered in relation to behavioural theory.

The Health Belief Model comprises six key constructs: perceived susceptibility, perceived severity, perceived benefits, perceived barriers, cues to action, and self-efficacy. These constructs were used to organize and interpret categories and sub-categories, particularly to understand how perceptions of risk, benefits, and barriers influenced engagement with maternal healthcare services. As described by Janz and Becker [28] individuals are more likely to take preventive health action when they perceive themselves to be at risk, believe the condition has serious consequences, and view the benefits of action as outweighing the barriers.

### Setting

The study was conducted at a hospital in Ghana that provides antenatal, intrapartum, and postnatal maternity services in the Ashanti region. The Ashanti Region has a high proportion of maternal deaths recorded yearly and is the most populous region in the country. In the hospital, antenatal care is primarily midwife-led, with a small team of midwives providing routine care throughout pregnancy. Women with uncomplicated pregnancies are reviewed by the same team of midwives for their scheduled antenatal visits until the onset of labour. If pregnancy-related complications are identified, women are referred to the hospital’s resident obstetrician for specialist assessment and management, after which care is shared between the obstetrician and the midwifery team for the remainder of the pregnancy. Labour and birth take place in the hospital’s labour ward and are attended by a separate team of midwives. The resident obstetrician becomes involved when obstetric complications arise or operative interventions, such as caesarean birth, are required. Women requiring specialist care beyond the district hospital’s capabilities, including complex obstetric or medical emergencies, are referred to the regional or teaching hospital. Following birth and discharge, women return to the antenatal clinic for routine postnatal follow-up and are reviewed by the same team of midwives who provided their pregnancy care. In Ghana, pregnant women are entitled to free or heavily subsidized antenatal, intrapartum and postnatal care through the National Health Insurance Scheme (NHIS), provided they are registered with the scheme. However, delays in NHIS reimbursements to health facilities may result in out-of-pocket expenses for women. In addition, some services and items, including certain medications and ambulance transport, may not be fully covered under the scheme.

### Participants and recruitment

Purposive sampling [29] was used to recruit participants at a health facility in the Ashanti region. Participants were recruited, and Eligible participants were pregnant women aged 20 years or older who were in their second trimester or later, attending the health facility, and not ill or admitted at the time of recruitment. Women were excluded if they were teenagers, attending for the first time, in the puerperal period, not attending the facility, or ill/admitted. Pregnant women were recruited from the antenatal clinic by the midwife in charge, who introduced the study to eligible participants. Written informed consent was obtained from all participants and documented on a signed consent form, witnessed by another midwife. Interviews were conducted after participants had completed their routine antenatal appointments at a location of their choosing. Most participants chose to be interviewed in a quiet, private area within the hospital grounds, typically under a shaded tree away from the antenatal clinic, where privacy could be maintained, and interruptions could be minimized. Two participants preferred to arrive before their scheduled antenatal appointment and were interviewed before receiving care.

### Data collection

All consenting pregnant women participated in confidential, face-to-face interviews conducted in English, using a semi-structured interview guide. Data were collected between 1 and 31 October 2021. Interviews were conducted by a female registered nurse undertaking her post-training clinical rotation at the study hospital. During her placement, she observed and assisted midwives in the antenatal clinic but was not involved in the clinical management or decision-making of participants. Although she was familiar with some participants through routine clinic interactions, she had no prior personal relationship with them. Her dual position as a nurse within the clinical setting was recognized as a potential influence on participants’ willingness to disclose their experiences or provide socially desirable responses. To minimize this influence, participants were explicitly informed that participation was voluntary, that declining or withdrawing would not affect their care, and that their responses would remain confidential. Interviews were conducted privately and without clinical staff present, and participants were encouraged to express their views openly.

The research team also recognized that its professional backgrounds and prior knowledge of the Health Belief Model (HBM) could influence the interpretation of participants’ accounts. To support reflexivity, the research team discussed how their clinical experience and prior use of the HBM might influence their interpretations and considered alternative readings of the data. To reduce the potential for HBM to shape participants’ responses or prematurely constrain interpretation, the initial analysis was conducted inductively before the HBM was applied deductively. This allowed codes, subcategories, and categories to be developed from participants’ accounts before examining their relationship with the HBM constructs [26].

The semi-structured interview guide was developed by the research team following a review of the literature on pre-eclampsia and maternal health-seeking behaviours [30]. The guide was designed to address the study aims through open-ended questions that explored participants’ understanding of pre-eclampsia, perceptions of who might be at risk, recognition of warning signs, and experiences accessing care. Standard questions were asked in the same order for all participants to ensure consistency, while allowing them to elaborate on issues of personal relevance. Examples of questions asked in the interviews included: *“Can you tell me what you know about pre-eclampsia?” What is your understanding of who might be at risk of developing pre-eclampsia? How do you feel about pre-eclampsia during your pregnancy? What concerns you most about it, if anything? What would you like your midwife to discuss with you about pre-eclampsia?* Probing questions were used as needed to clarify responses and guide the discussion.

During the interview, participant confidentiality was maintained by using pseudonyms. The interviews explored participants’ socio-demographic characteristics and experiences related to pregnancy and care-seeking, with personal identifiers such as names excluded to maintain confidentiality. Interviews lasted approximately 18–30 minutes. Data saturation was defined as the point at which additional interviews no longer generated substantially new information relevant to the research questions. Data saturation was assessed through preliminary review and discussion of the interview data by authors IG, EF, EOA. Following the 14th interview, the research team observed that participants’ accounts were becoming increasingly repetitive, with no substantially new information emerging regarding the study objectives. Two additional interviews were conducted to assess whether further insights would emerge. These interviews did not identify substantially new information, supporting the team’s assessment that data saturation had been reached. Formal data analysis was undertaken after data collection was completed and was not conducted concurrently with the interviews. This approach is consistent with recommendations that saturation be assessed iteratively throughout data collection and analysis rather than determined solely by a predetermined sample size [31].

### Data analysis

Data were analyzed using qualitative content analysis following an iterative process of moving from participants’ accounts to increasingly abstract levels of interpretation [30]. Qualitative content analysis [30,32] was selected due to its flexibility and the systematic process in identifying, organizing and interpreting data. Particularly, Graneheim and Lundman’s method was followed [27]. Audio-recorded interviews were reviewed, validated, and transcribed immediately after data collection to ensure accuracy and minimize loss of contextual detail. Transcripts were uploaded into NVivo version 12 to facilitate systematic data management and analysis.

The analysis began with repeated readings of the transcripts to become familiar with the data and to identify content areas relevant to the study objectives, from which meaning units relevant to the research questions were identified. Meaning units were condensed while preserving their central meaning and were subsequently assigned codes that captured their substantive content. Related codes were compared for similarities and differences and grouped into subcategories, which were further abstracted into broader categories [30]. Thus, the analytic progression was meaning units → condensed meaning units → codes → subcategories → categories. Throughout the analytical process, the transcripts, meaning units, codes, subcategories and categories were revisited to ensure that the interpretations remained grounded in participants’ accounts. This process enabled the researchers to remain grounded in participants’ accounts while progressively developing higher-level descriptions of the phenomenon.

A hybrid inductive–deductive approach was used to integrate data-driven analysis [26] with the Health Belief Model. The initial coding and development of subcategories and categories were primarily inductive, allowing findings to emerge from participants’ accounts without imposing HBM constructs on the data. Following development and refinement of the categories, the categories were examined in relation to the HBM constructs to identify how participants’ accounts reflected perceived susceptibility, perceived severity, perceived benefits, perceived barriers, cues to action, and self-efficacy [S1 Table]. The HBM, therefore, served as a theoretical interpretive framework applied after the primary content analysis [33], rather than as the mechanism through which the initial categories were generated. All components of HBM were clearly identified during data analysis, yielding 13 categories that aligned with an HBM construct; no categories were excluded or forced into the framework.

### Ethical considerations

The study received ethical approval from the XXX Hospital Committee on Human Research, Publication, and Ethics (CHRPE/AP/097/21). Permission to conduct the study was obtained from the participating healthcare facility. Informed consent was obtained from all participants after they had received detailed information about the study. Participants were informed that participation was voluntary, that refusal would not affect the care they received, and that all data would be handled confidentially.

### Trustworthiness

Several strategies were used to enhance the trustworthiness of the findings, including credibility, dependability, confirmability, reflexivity, and transferability [34]. Data collection continued until saturation was reached, with 14 interviews completed, followed by two additional interviews confirming that no substantially new information or categories were emerging. Interviews lasted approximately 18–30 minutes and were audio-recorded with participants’ consent and transcribed verbatim. These procedures provided a consistent and detailed record of participants’ accounts and supported credibility and confirmability [34]. Also, four researchers involved in the analysis were midwives with more than five years of professional experience. As health professionals familiar with the study setting, and with prior experience using the HBM in research, they brought clinical, contextual and theoretical knowledge to the study. The team recognised that this familiarity could shape the questions asked, the meanings identified in participants’ accounts and their interpretation [35]. To limit the influence of the HBM on the initial analysis, categories were developed inductively before being considered in relation to its constructs.

For dependability, the analytic process was conducted systematically, with two researchers independently reviewing and coding the interview data. Differences in interpretation were discussed and resolved through consensus, with a third researcher involved when necessary. Researchers also used analytic memoing to document emerging interpretations and decisions throughout the analysis [34]. These procedures created an audit trail of the analytic process and helped maintain consistency between the data, codes, and final findings. Transferability was supported through purposive recruitment of participants with relevant experiences and detailed reporting of the study setting, participants, data collection procedures, and analytic process [35]. Rather than assuming statistical generalizability, these contextual details allow readers to assess the applicability of the findings to populations or settings with similar characteristics. Reflexive engagement with the data and repeated review of transcripts, codes, and categories further supported the credibility and interpretive depth of the findings.

## Results

A total of 16 women participated in the study. Participants were aged between 22 and 32 years, with the majority in their mid-to-late twenties. Most participants were married [n = 14], with only two identifying as single. Educational attainment varied, with many participants having completed senior high school (SHS). Participants were engaged in a range of occupations, including trading, teaching, fashion design, and hairdressing. A small number were unemployed. In terms of obstetric history, participants ranged from primigravida (G1P0) to multiparous women (G4P3), reflecting a mix of first-time and experienced mothers.

### Perceived susceptibility

Participants showed a range of perceptions about their risk of developing pre-eclampsia, shaped by personal health factors, previous experiences, and community narratives. Some perceived low risk due to age, health status, or absence of family history, while others, particularly those with prior experience of pre-eclampsia, expressed greater concern about recurrence.

#### Low perceived risk of pre-eclampsia.

Many participants perceived themselves to be at low risk of pre-eclampsia, citing good health and the absence of known personal or familial risk factors. Although some were aware of general risk factors, they did not identify with them, and a limited understanding of familial risk further reinforced low perceived susceptibility.

*“I’ve always had healthy pregnancies, and I don’t have any medical conditions, so I don’t think pre-eclampsia can happen to me. I feel like it’s something for women with health problems, not someone like me”* **P6**

Another participant explained:

*“The nurses say women who are older or have high blood pressure are at risk, but I’m only 24…….I don’t think I am at risk for it”* **P8**

Others believed that because no one in their family had experienced complications, they were unlikely to be affected:

*“In my family, nobody has ever had high blood pressure during pregnancy. Because of that, I don’t think it will happen to me either”* **P15**

#### Perceived risk from observing others’ experiences.

In contrast to participants who perceived themselves at low risk based on personal health and family history, others reported heightened perceived susceptibility shaped by observing pre-eclampsia in close friends or relatives. Vicarious experiences heightened both fear and awareness, even among women without identifiable risk factors, and shaped their engagement with antenatal care and self-monitoring practices.

*“I have never had pre-eclampsia myself, but I watched my close friend suddenly develop it during her second trimester last year. She was always healthy, and it came out of nowhere. Seeing that made me worried that it could happen to me too, so now I pay extra attention to my blood pressure and every little symptom”* **P16**

#### Community narratives influencing perceived vulnerability.

Participants’ perceptions of vulnerability to pre-eclampsia were shaped by community narratives and shared experiences, reinforcing the belief that anyone could be at risk.

*“Some women told me different things about pre-eclampsia …. Listening to them [other women] makes me feel like I might also be at risk.”* **P9**

These experiences not only raised awareness of susceptibility but also prompted some participants to reflect on factors that might trigger the condition, such as stress or workload.

*“Some of my colleagues at work were always careful with their diet and rested well, yet they still developed BP when they were pregnant…….even small stresses or hard work could put me at risk, so I try to take breaks and avoid heavy chores”* **P12**

### Perceived severity

Perceived severity reflects participants’ beliefs about how serious and potentially life-threatening pre-eclampsia can be for both the mother and the baby. Participants generally perceived the condition as dangerous, often accompanied by fear, emotional distress, and concern for pregnancy outcomes.

#### Fear of maternal death or severe complications.

Fear of maternal death or severe complications emerged as a prominent concern among participants. This anticipatory fear was shaped by accounts of other women’s experiences and messages received during antenatal health education, with pre-eclampsia commonly described as frightening, potentially fatal, and deeply affecting women’s lives

*“Eieee, sister, the pre-eclampsia is no joke, a lady attending my church died because of it. It was a very painful incident because she was a newlywed”* **P11**

These fears were further reinforced by participants’ understanding of the medical consequences associated with pre-eclampsia.

*“The nurses explained to us that it can cause problems like seizures or damage to vital organs, so anytime I feel unwell, I start worrying that it may lead to something serious”* **P2**

#### Fear of pre-eclampsia affecting the baby’s health.

Participants feared that pre-eclampsia could harm fetal health, contributing to increased anxiety during pregnancy.

*“The nurses told us that pre-eclampsia can affect the baby, especially the baby’s growth, so even though my baby was still moving, I was always worried that something might be happening inside”* **P7**

These concerns were further influenced by participants’ understanding of how pre-eclampsia could compromise the baby’s health before birth.

*“They explained that high blood pressure can reduce the blood going to the baby, so I was scared that my baby might not be getting enough nutrients or oxygen”* **P10**

### Perceived benefits

Perceived benefits reflected women’s confidence that timely care-seeking, adherence to recommended practices, and lifestyle changes can help prevent or manage pre-eclampsia and improve maternal and fetal outcomes.

#### Perceived benefits of formal antenatal and professional healthcare.

Participants saw regular antenatal care as essential for early detection, timely management, and reassurance, strengthening trust in formal maternal healthcare.

*“Whenever I come for antenatal clinic, I feel safer because the nurses check my blood pressure and explain what I should do, so I believe coming to the clinic helps prevent serious problems”* **P8**

Participants preferred expert medical care for pre-eclampsia, valuing professional monitoring and intervention, though some concerns remained, and non-hospital care was seen as risky.

*“I prefer to be cared for by doctors and nurses because they understand pre-eclampsia better and know what to do if any complication comes up”* **P14**

Some participants described family pressure to use traditional birth attendants, which they strongly resisted due to concerns about delayed care, reflecting women’s agency in care seeking.

*“My mother-in-law wants me to use a traditional birth attendant, but I refused because they may not even know what is wrong until it is too late. I decided I would rather go to a well-equipped healthcare facility with doctors and nurses”* **P6**

#### Belief in the usefulness of lifestyle and dietary changes.

Participants believed that lifestyle and dietary changes can help manage pre-eclampsia and reduce risks and viewed antenatal advice as practical steps to protect their health and their babies.

*“I had to stop doing heavy work and rest more, even when people around me did not understand why, because I was told stress and overworking could worsen my condition”* **P2**

Other participants viewed dietary and lifestyle changes as protective actions taken despite uncertainty about immediate outcomes.

*“Even though I cannot see the effect immediately, I still follow the diet and rest advice strictly because I was told pre-eclampsia can become serious suddenly, and I don’t want to ignore what might help prevent problems for my baby and me”* **P15**

### Perceived barriers

Perceived barriers reflected challenges that limited participants’ ability to seek, access, or continue appropriate care for pre-eclampsia. These barriers included financial, social, and health system constraints that shaped care-seeking and engagement with maternal health services.

#### Personal level barriers.

Financial pressures, including the cost of transportation, clinic visits, medications, and diagnostic tests, often compete with household needs, leading to delayed care or consideration of lower-cost alternatives.

*“Most times I want to attend the clinic, but when I calculate the transport money, the tests they will ask me to do, and the drugs I might be given, it becomes overwhelming because I don’t always have that kind of money available”* **P16**

Practical challenges such as long travel distances, unreliable transport, and childcare responsibilities further hindered timely clinic attendance.

*“I have three children at home, and it is difficult to leave them alone. I must arrange for my mother or neighbour to take care of them whenever I want to come to the hospital, and sometimes no one is available”* **P12**

Very few complained about the distance of the facility from their houses, which sometimes makes it difficult to access care:

*“The facility for my antenatal is quite far from my place, and this sometimes discourages me”* **P9**

Reliance on husbands or family for permission reduced women’s autonomy, delaying care and forcing them to balance health needs with family expectations and social hierarchies.

*“My mother-in-law insists that I should follow what she thinks is right, and I cannot go to the clinic without her agreement. I must respect her decision….It makes me anxious, knowing that my health depends on someone else’s decision”* **P4**

#### Mistrust and inadequate communication in hospitals.

Fear of hospitals, negative past experiences, and mistrust of medical staff, often shaped by personal encounters, shared stories, and cultural beliefs, emerged as barriers to timely and consistent care-seeking.

*“After what happened to my friend at the hospital, I became skeptical. She went for delivery and had so many problems that could have been handled differently, and I don’t want to go through that”* **P6**

One woman shared that although she recognized the hospital as safer, previous negative experiences, particularly being shouted at when asking questions, left her feeling anxious about returning.

*“Even though I know the hospital is safer, I still feel scared because in the past I was shouted at when I asked questions, and it made me anxious about going back”* **P10**

These concerns were compounded by poor communication from healthcare workers, including unclear or rushed explanations, which undermined understanding, increased anxiety, and weakened trust in maternal healthcare services, ultimately affecting adherence to medical advice.

*“Sometimes the nurses just tell you that your blood pressure is high or that something is wrong, but they don’t explain what it really means or what I should do. I leave the clinic feeling confused and worried”* **P15**

One participant reported her questions about routine medications were dismissed, reflecting poor communication and limited patient engagement

*“I asked about the routine medications they gave me because this is my first time being pregnant, and the nurse quickly said I should take them without really telling me what they do”* **P3**

### Cues to action

Participants described how bodily sensations and guidance from healthcare providers and social networks prompted vigilance and proactive engagement with maternal care, supporting timely recognition and response to pre-eclampsia.

#### Previous experience as a cue to action.

Previous experience with pre-eclampsia symptoms improved recognition of warning signs, increased vigilance, and prompted earlier care-seeking in subsequent pregnancies.

*“During my first pregnancy, I had severe swelling in my legs and high blood pressure, and I ended up being admitted. This time, whenever I notice my hands or feet swelling, I immediately measure my blood pressure and go to the clinic without waiting”* **P11**

A few participants also reported that prior experiences enabled them to notice warning signs in other women and also monitor their own health.

*“Having gone through pre-eclampsia before, I can spot the signs… last week I saw a church member of mine with swollen legs, hands and feet, and she also complained of headaches. I then advised her to go to the clinic”* **P16**

#### Professional guidance prompting timely action.

Clear advice and personalized risk information from trusted healthcare providers increased urgency and prompted timely care-seeking.

*“The doctor told me clearly that if my blood pressure rises or I notice swelling in my face or hands, I must come immediately, otherwise it could be dangerous for the baby and me. That really stayed with me, and I act on it without delay”* **P9**

Participants found professional guidance, particularly antenatal health talks, useful in prompting them to take appropriate action when concerns arose.

*“The health talks at the clinic really opened my eyes. I learned about the signs that could indicate danger”* **P3**

### Self-efficacy

Self-efficacy reflected participants’ application of antenatal knowledge, enabling them to better manage pregnancy-related challenges. They described monitoring their blood pressure at home, keeping records of blood pressure readings from antenatal visits, paying attention to recognized warning signs, communicating symptoms to healthcare providers, and drawing on family support to help navigate their care.

*“I pay attention to even mild headaches or dizziness now because I know these could be early signs”* **P16**

One participant explained that she felt confident in reporting her symptoms and drew on her family’s support to navigate her care.

*“I don’t hesitate to share any unusual symptoms with my mother. She reminds me about tests or medications I might forget”* **P8**

Participants demonstrated self-efficacy by actively monitoring their pregnancy health through practical self-monitoring strategies.

*“I write down all the advice from each clinic visit in a notebook…… I even learned to record my daily BP checks in a book and take it along with me to the clinic”* **P2**

## Discussion

This study explored Ghanaian women’s health beliefs about pre-eclampsia and how these shape care-seeking. Risk perceptions were varied and socially influenced, with high perceived severity and strong belief in the protective effect of antenatal care. However, poor healthcare interactions, communication gaps, and structural and relational barriers limited timely care-seeking despite intentions. Our findings are consistent with those of a Nigerian study, which similarly showed that women’s perceived susceptibility and care-seeking behaviours are shaped by a combination of social influences, healthcare experiences, and the quality of information received during antenatal care [12]. Similarly, others have also reported ineffective communication [36], along with women’s geographic location, socioeconomic conditions, and fear of mainstream health facilities, can influence care-seeking behaviour [20].

While participants generally recognized the seriousness of pre-eclampsia, some demonstrated low perceived susceptibility, often believing they were not personally at risk in the absence of symptoms or identifiable risk factors. This is concerning, as low perceived risk and misconceptions may delay engagement with preventive behaviours, reduce vigilance in symptom monitoring, and contribute to late presentation [4]. Within the Health Belief Model, perceived susceptibility is a key driver of action; therefore, underestimation of personal risk can weaken motivation to seek timely care, even when awareness of severity is high [28]. These findings highlight the relevance of maternal health literacy in shaping health beliefs related to pre-eclampsia. Further, it underscores the need for antenatal education to move beyond general awareness and emphasize personalized risk communication, ensuring that all women, regardless of perceived risk, remain alert to early warning signs and the need for prompt care-seeking. This is particularly important in contexts such as Ghana, where low levels of knowledge about pre-eclampsia have been reported, suggesting persisting gaps in maternal health literacy [18,24]. Strengthening women’s health literacy is therefore essential to support timely recognition and appropriate response [4,37]. However, greater risk awareness should be accompanied by effective risk communication to promote informed decision-making without causing unnecessary anxiety [38].

These findings also suggest, as others have, that broader health system factors shape how intention translates into action [39]. The presence of structural and relational barriers, such as poor communication, negative care experiences, and limited resources, is a hindrance to women’s ability to act promptly despite their awareness and willingness [18,36,40]. In Ghana, antenatal care attendance is high and even so, in pre-eclampsia [18,19,36], but respectful care is often compromised, particularly when health workers perceive women as non-compliant [41]. This highlights the need for structural reform and more responsive care in line with WHO recommendations [42]. Health information should be tailored to women’s individual contexts, and culturally appropriate communication tools can reinforce key messages across the continuum of antenatal care.

Focused community-based interventions, such as transport and heavily subsidized medication and care, have strong potential to improve pre-eclampsia outcomes [43]. Efforts to reduce systemic impediments can enable timely access, support continuity of care, and ensure that women can act on clinical advice without financial or logistical constraints. The Ghana National Health Insurance Scheme provides coverage for antenatal, labour, and postnatal care. However, delays and inadequacies in reimbursement, limited benefit coverage, and frequent stockouts undermine its effectiveness [44]. These challenges necessitate a review of the policy to ensure it comprehensively covers all relevant costs, particularly those associated with direct causes of maternal mortality such as pre-eclampsia. Other interventions, such as maternal health helplines, mHealth, and telehealth, are scalable and have great potential to support timely decision-making and more appropriate care-seeking. A study conducted in Ghana demonstrated the effectiveness of an on-call phone line providing clinical advice to pregnant women with home-monitored high blood pressure [45]. Such models could be adapted to complement antenatal care. However, its scalability may vary across regions depending on infrastructure, access to mobile technology, and health system capacity [36].

### Strengths and limitations

This study provides context-specific insights into women’s perceptions of pre-eclampsia through the Health Belief Model, offering a deeper understanding of how perceived susceptibility and severity, cues to action, and self-efficacy influence health behaviours. The qualitative descriptive approach allowed participants’ accounts to inform the categories before these were interpreted using the Health Belief Model. This helped retain experiences grounded in the study setting while identifying how HBM constructs may inform education and care. Additionally, the study contributes to the limited body of literature on perceptions of pre-eclampsia in low-resource settings, particularly in the Ghanaian context, thereby offering context-specific evidence to inform targeted interventions.

Participants were recruited from the general population of pregnant women rather than specifically from women with prior experience of pre-eclampsia. The findings may therefore offer less insight into how experiencing the condition shapes perceptions and decisions. However, the findings are situated within the specific social, cultural, and healthcare context of the study setting and should therefore be considered in relation to their potential transferability to other populations or settings. As the study relied on participants’ accounts, descriptions of past experiences may be subject to recall bias, and participants may have been reluctant to express views they considered socially undesirable. The researchers’ clinical backgrounds and familiarity with HBM may also have influenced interpretation, although inductive category development before applying the model helped address this.

## Conclusion

This study highlights the perceived health beliefs of women regarding pre-eclampsia, a dangerous pregnancy complication. These findings underscore shifting from standardized antenatal education to personalized, risk-based antenatal counselling, supported by clear communication within a respectful maternity care framework to strengthen women’s confidence and timely care-seeking. Culturally appropriate education on pre-eclampsia should be integrated into routine antenatal care. At the policy level, addressing financial and structural barriers, strengthening provider training in communication and respectful care, and investing in community and mass media education are critical. Overall, improving pre-eclampsia outcomes requires a coordinated approach that aligns quality clinical care with supportive health system policies.

## Supporting information

S1 Table Analytical table. (DOCX)
