# Supplementary material for: Pregnant women’s health beliefs about pre-eclampsia in a resource-limited setting: A qualitative study
Source: PLoS One. 2026 Oct 30;21(10):e0361626. doi: 10.1371/journal.pone.0361626 (PMC13722199; doi:10.1371/journal.pone.0361626)
Supplement: Supplementary file 1 [file pone.0361626.s001.docx]

Supplementary Table S1: Analytical table

| **Analytical level** | **Example 1** | **Example 2** | **Example 3** |
| --- | --- | --- | --- |
| Meaning unit | “I’ve always had healthy pregnancies…” (P6) | “My neighbor developed preeclampsia unexpectedly…” (P11) | “I hear stories from older women…” (P5) |
| Condensed meaning unit | Previous healthy pregnancies reduce perceived personal risk | Observing preeclampsia in others increases perceived risk | Community stories influence risk perceptions |
| Code | Low risk due to good health | Risk awareness through others' experiences | Influence of community narratives |
| Subcategory | Personal health and risk perception | Observing others' experiences | Community experiences |
| Category | Low perceived personal risk | Perceived risk from observing others | Community narratives influencing vulnerability |
| HBM construct | Perceived susceptibility | Perceived susceptibility | Perceived susceptibility |

Note. The table presents selected examples illustrating the progression from meaning units to condensed meaning units, codes, subcategories and categories, following Graneheim and Lundman's qualitative content analysis approach. Categories were developed inductively before being examined in relation to the constructs of the Health Belief Model.
